# Chemical modification of polyvinyl chloride and silicone elastomer in inhibiting adhesion of *Aeromonas hydrophila*

**DOI:** 10.1007/s11274-013-1282-8

**Published:** 2013-02-10

**Authors:** Dorota Kregiel, Joanna Berlowska, Urszula Mizerska, Witold Fortuniak, Julian Chojnowski, Wojciech Ambroziak

**Affiliations:** 1 Institute of Fermentation Technology and Microbiology, Technical University of Lodz, ul. Wolczanska 171/173, 90-924 Lodz, Poland; 2 Center of Molecular and Macromolecular Studies, Polish Academy of Sciences, ul. Sienkiewicza 112, 90-363 Lodz, Poland

**Keywords:** *Aeromonas hydrophila*, Biofilm, Polyvinyl chloride, Silicone elastomer, Organo-silanes

## Abstract

Disease-causing bacteria of the genus *Aeromonas* are able to adhere to pipe materials, colonizing the surfaces and forming biofilms in water distribution systems. The aim of our research was to study how the modification of materials used commonly in the water industry can reduce bacterial cell attachment. Polyvinyl chloride and silicone elastomer surfaces were activated and modified with reactive organo-silanes by coupling or co-crosslinking silanes with the native material. Both the native and modified surfaces were tested using the bacterial strain *Aeromonas hydrophila*, which was isolated from the Polish water distribution system. The surface tension of both the native and modified surfaces was measured. To determine cell viability and bacterial adhesion two methods were used, namely plate count and luminometry. Results were expressed in colony-forming units (c.f.u.) and in relative light units (RLU) per cm^2^. Almost all the chemically modified surfaces exhibited higher anti-adhesive and anti-microbial properties in comparison to the native surfaces. Among the modifying agents examined, poly[dimethylsiloxane-co-(*N*,*N*-dimethyl-*N*-*n*-octylammoniopropyl chloride) methylsiloxane)] terminated with hydroxydimethylsilyl groups (20 %) in silicone elastomer gave the most desirable results. The surface tension of this modifier, was comparable to the non-polar native surface. However, almost half of this value was due to the result of polar forces. In this case, in an adhesion analysis, only 1 RLU cm^−2^ and less than 1 c.f.u. cm^−2^ were noted. For the native gumosil, the results were 9,375 RLU cm^−2^ and 2.5 × 10^8^ c.f.u. cm^−2^, respectively. The antibacterial activity of active organo-silanes was associated only with the carrier surface because no antibacterial compounds were detected in liquid culture media, in concentrations that were able to inhibit cell growth.

## Introduction

The World Health Organization lists *Aeromonas hydrophila*, a member of the genus *Aeromonas,* as a potential waterborne pathogen (WHO 2006). *Aeromonas* spp. can cause diarrhea, wound infections, septicemia, meningitis, ophthalmitis, endocarditis, aspiration pneumonia, and biliary tract infections, most frequently affecting young children (under 5 years of age) and older adults (over 60 years of age). The number of cases of *Aeromonas*-associated gastroenteritis increases during the summer and correlates with an increased number of aeromonads in water systems (US EPA 2006; Kregiel and Rygala 2010).

Species of *Aeromonas* can commonly be found in a variety of aquatic environments, including freshwater, estuarine, brackish, and salt waters. The percentage of *A. hydrophila* among heterotrophic microorganisms in Polish surface waters ranges from 6 to 22 % (Golas et al. 2009). *Aeromonas* bacteria are a normal part of the microflora found in these waters, and their presence does not indicate that the water has been polluted. As *Aeromonas* spp. are common in natural waters, and because it is generally accepted that some strains can be pathogenic to humans, these bacteria seem to be indicators of the general decline in the bacteriological quality of water in distribution systems.

*Aeromonas* spp. can have different adhesive abilities, depending on environmental conditions (Pianetti et al. 2012). A single polar flagellum facilitates both adhesion and invasion of human and fish cell lines. In viscous environments or over surfaces, aeromonads are able to produce many peritrichous lateral flagella, which increase bacterial adhesion and are required for biofilm formation (Saidi et al. 2011). Other studies suggest that non-pillar polysaccharide adhesins also play an important role in adhesion of *Aeromonas* spp. (Bonet et al. 1993).

It has been widely accepted that environmental conditions (temperature, pH, chlorine, ionic strength, electrolyte type), as well as interfacial factors (surface charge, surface energy, chemical nature) and physiological factors (microorganism type, its growth stage, and metabolic activity) control the formation and properties of biofilms (Srinivasan et al. 2008; Kregiel and Rygala 2010). Among these factors, only solid surface properties can be changed artificially to prevent biofouling in water distribution systems (Li et al. 2010).

Common materials used in drinking water distribution systems belong to thermoplastics, including polyvinyl chloride (PVC), rubbers, and silicones (Tomboulian et al. 2004). These are universal polymers which also can be used in the production of a wide variety of materials. PVC can be processed into short-life products such as packaging materials used in the food and water industries (Sadat-Shojai and Bakhshandeh 2011). Silicone elastomers are popular in food manufacturing and processing, and in medicine in peristaltic pumps, aspirating, suction, and infusion devices.

A significant number of studies on biofilm prevention have focused on surface modifications. Traditional techniques involve designing coatings with different biocidal agents, including antibiotics, quaternary ammonium salts, and silver. However, these compounds of relatively low molecular weight may be highly toxic, and can have mutagenic and/or carcinogenic properties. In order to overcome these problems, water-insoluble antibacterial materials, in which antibacterial groups have been chemically immobilized on water-insoluble carriers, have been developed (Gao et al. 2008). There is a vast number of compounds which have biocidal groups chemically bonded to their chains. They can effectively inhibit the growth of microorganisms without releasing toxic products of low molecular weight into the environment. When attached covalently to the surfaces, they are able to kill bacteria on contact, and their antimicrobial activity is durable and sustainable. Biocidal compounds with polycationic structures, such as those containing quaternary ammonium salt or alkyl groups, can be used as additives in a wide variety of applications, in order to modify surface properties including surface tension, surface charge, hydrophobicity/hydrophilicity, and adhesiveness (Siedenbiedel and Tiller 2012). Such compounds may include organo-silanes, which contain at least one silicon-carbon bond, for instance Si–CH_3_. The carbon-silicon bond is very stable and nonpolar, and the presence of an alkyl group produces changes in surface tension, and gives improved hydrophobic qualities (Andriot et al. 2009). Additionally, organo-silanes may contain different types of reactive antibacterial groups, such as methoxy, ethoxy, acetoxy, epoxy, amino, methacryloxy, or sulfido groups.

In this research, we compared the antibacterial and antiadhesive activity of various PVC and silicone elastomer materials after having modified them using different active organo-silanes. Both the native and modified surfaces were tested using the bacterial strain *A. hydrophila* LOCK0968.

## Materials and methods

### Bacterial culture and media

The bacterial strain *A. hydrophila* LOCK0968 from Culture Collection 105 (Lodz, Poland) was isolated from the unchlorinated water distribution system in Poland (Kregiel and Rygala 2010). The strain was stored on antibiotic agar (Merck, Germany) slants at 4 °C in standard laboratory conditions.

*A. hydrophila* cells were grown in an antibiotic broth (Merck) at 25 °C for 24 h. Bacterial cells from the liquid cultures were harvested by centrifugation (15 min, 6,000×*g*, 5 °C), washed twice, and suspended in 100 ml of sterile water to~9 × 10^8^ c.f.u. ml^−1^ by comparison with a McFarland no. 3 turbidity standard (Densitometer DEN-1, Grant). Finally, the bacterial suspension was diluted to approximately 9 × 10^2^ c.f.u. ml^−1^.

For the aerobic cultures, 50-fold diluted buffered tryptone water (Merck) with a concentration of 200 mg l^−1^ of peptone was prepared and poured (20 ml) into 25 ml Erlenmeyer flasks. After sterilizing the culture medium, the inoculum (2.0 ml) of the bacterial strain and a sterile carrier were added to each flask. The initial cell concentration in the culture medium was 10^1^–10^2^ c.f.u. ml^−1^. The samples were incubated at 15 °C on a laboratory shaker (200 rpm) for 10 days.

### Native surfaces study

**Fig. 1:**
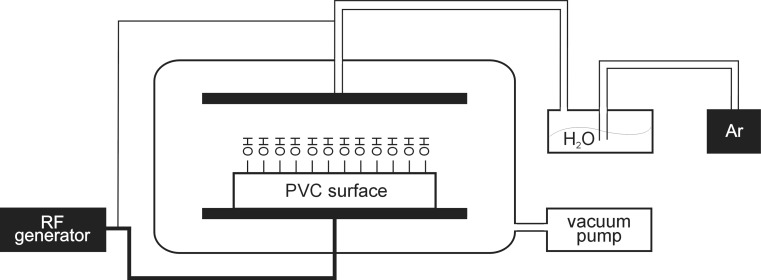
Scheme of plasma generator used for PVC surface activation

Two native surfaces, namely polyvinyl chloride (PVC) and silicone elastomer (gumosil, G) materials, as well as their active modifications, were used in the study. Polyvinyl chloride (PVC) and silicone elastomer (G) surfaces were activated and modified using reactive organo-silanes. The native PVC carriers were formed from ORIPLAST U granulate (ORIANEX Ltd., Poland). The modifications of PVC surfaces were made by coupling silanes with the native material. At the beginning of the PVC modification procedure, the plates were irradiated by radio frequency generated plasma using the apparatus shown schematically in Fig. 1. The conditions of the experiments were as follows: pressure 300 Pa, power 40 W, time of exposure 2 min. During this stage of the process, a large number of –OH groups (1.47 nmol cm^−2^) were generated on the surface of the carrier. During the second part of the procedure, the activated PVC plate was immersed for 24 h in 900 ml of organo-silane solution (2 × 10^−3^ mol l^−1^), containing 2 × 10^−8^ mol of tin (II) octoate as the catalyst. Then, the plate was washed with toluene (100 ml) and dioxane (100 ml). The modifications of the silicone elastomer were made by co-crosslinking organo-silane with an RTV material. Linear PDMS “Gumosil” (Mn = 33,000) was provided by the chemical company “Silikony Polskie” (Nowa Sarzyna, Poland). A mixture of tetraethoxysilane (TEOS) and dibutyltin dilaurate (DBTDL) was used in the co-crosslinking procedure (Fortuniak et al. 2011). All the native materials and their modifications are listed in Table 1. The carrier plates (size 60 × 20 mm) were sterilized in 70 % ethanol for 1 h, followed by UV irradiation (265 nm) for 1 h on each side. The carrier plates (size 60 × 20 mm) were sterilized in 70 % ethanol for 1 h, followed by UV irradiation (265 nm) for 1 h each side.

**Table 1:** Native surfaces and their chemical modifications

Native surface	Modification		
	Symbol	Active compound coupled to –OH groups (1.47 nmol cm^−2^)	Formula
Polyvinyl chloride PVC	P1	1H,1H,2H,2H-Perfluorooctylmethyldimethoxysilane	
	P2	Isobutylmethyl dimethoxysilane	
	P3	(3-Glycidoxypropyl)trimethoxysilane	
	P4	3(1-Tertbutylamine-2-hydroxy)propyloxypropyl diethoxymethylsilane	

	Symbol	Cross-linked active compound (% in matrix)	Formula
Silicone elastomer GUMOSIL	G1	poly[dimethylsiloxane-co-(*N*,*N*-dimethyl-*N*-*n*-octylammoniopropyl chloride)methylsiloxane)] terminated with hydroxydimethylsilyl groups (20 %)	
	G2	poly[dimethylsiloxane-co-(*N*,*N*-dimethyl-*N*-*n*-octylammoniopropyl chloride)methylsiloxane)] terminated with hydroxydimethylsilyl groups (10 %)	
	G3	poly[dimethylsiloxane-co-(*N*,*N*-dimethyl-*N*-*n*-hexadecylammoniopropyl chloride) methylsiloxane)] terminated with hydroxydimethylsilyl groups (20 %)	
	G4	poly[dimethylsiloxane-co-(*N*,*N*-dimethyl-*N*-*n*-hexadecylammoniopropyl chloride) methylsiloxane)] terminated with hydroxydimethylsilyl groups (10 %)	

### Adhesion analysis

In order to identify the changes that occurred on the modified surfaces, the contact angle for the tested materials was taken. The determination of the contact angle values for the two solvents, a dispersive one (dimethylformamide, DMF) and a polar one (water), enabled calculation of the surface energy. All measurements were performed using the RAME HART NRL goniometer equipped with CAMERA JVC KYF 70B. The dynamic contact angle was calculated using the DROP program and given as an average of about 15 measurements (Fortuniak et al. 2011).

An analysis of bacterial adhesion to the carriers involved luminometric measurements, a colony count method and microscopic observations followed by a qualitative assessment of the density of bacteria covering the plates (performed on tenth day of the experiment). For the luminometric tests, the carrier plate was removed from the culture medium, rinsed with sterile distilled water and swabbed by HY-LiTE2 sampling pens (Merck) for surface testing. The measurement was performed in relative light units (RLU) by the HY-LiTE2 luminometer (Kregiel and Rygala 2010).

For the colony count, the carrier plate was removed from the culture medium, rinsed with sterile distilled water and swabbed with sterile swabs for surface testing. The bacterial suspensions were transferred onto PCA agar medium (Merck), and after incubation (25 °C, 48 h) the colonies were counted. The number of colony forming units per ml of the medium and per cm^2^ of the carrier was calculated and presented as a logarithmic function. In the microscopic studies, *A. hydrophila* cells were stained with basic fuchsin (0.5 %) and observed using a light microscope (OLYMPUS type BX41) fitted with a 50 × lens and with top illumination of the tested surfaces by an external lamp. Images were captured with a digital camera and analyzed using the UTHSCA ImageTool program available from http://ddsdx.uthscsa.edu/dig/itdesc.html.

### Antibacterial activity of the culture medium

The antibacterial activity of the culture medium was examined after a 10-day incubation of the carrier in the sterile culture medium. For this assessment, the Kirby-Bauer test, known as the disk-diffusion method, was used (Jorgensen and Turnidge 2007). This method involves the inhibition of bacterial growth measured under standard conditions. For this test, a culture medium, TSA agar (Merck), was uniformly and aseptically inoculated with the *A. hydrophila* strain. Sterile filter paper discs (ϕ = 5 mm), which had been previously impregnated with the culture medium after the 10-day incubation with the carrier, were then placed on the agar medium. After a 24-h incubation period at 25 °C, if the active compound was present in a concentration inhibiting bacterial growth, a clear zone around the paper disc could be observed.

### Statistics

Mean values were calculated from the data obtained from the three independent experiments. Comparisons between the mean values were performed using an analysis of variance: one-way ANOVA followed by post hoc Tukey’s test (STATISTICA 10, StatSoft^®^ Poland). Statistical significance was set at the conventional level of 5 % (*p* < 0.05).

## Results

**Fig. 2:**
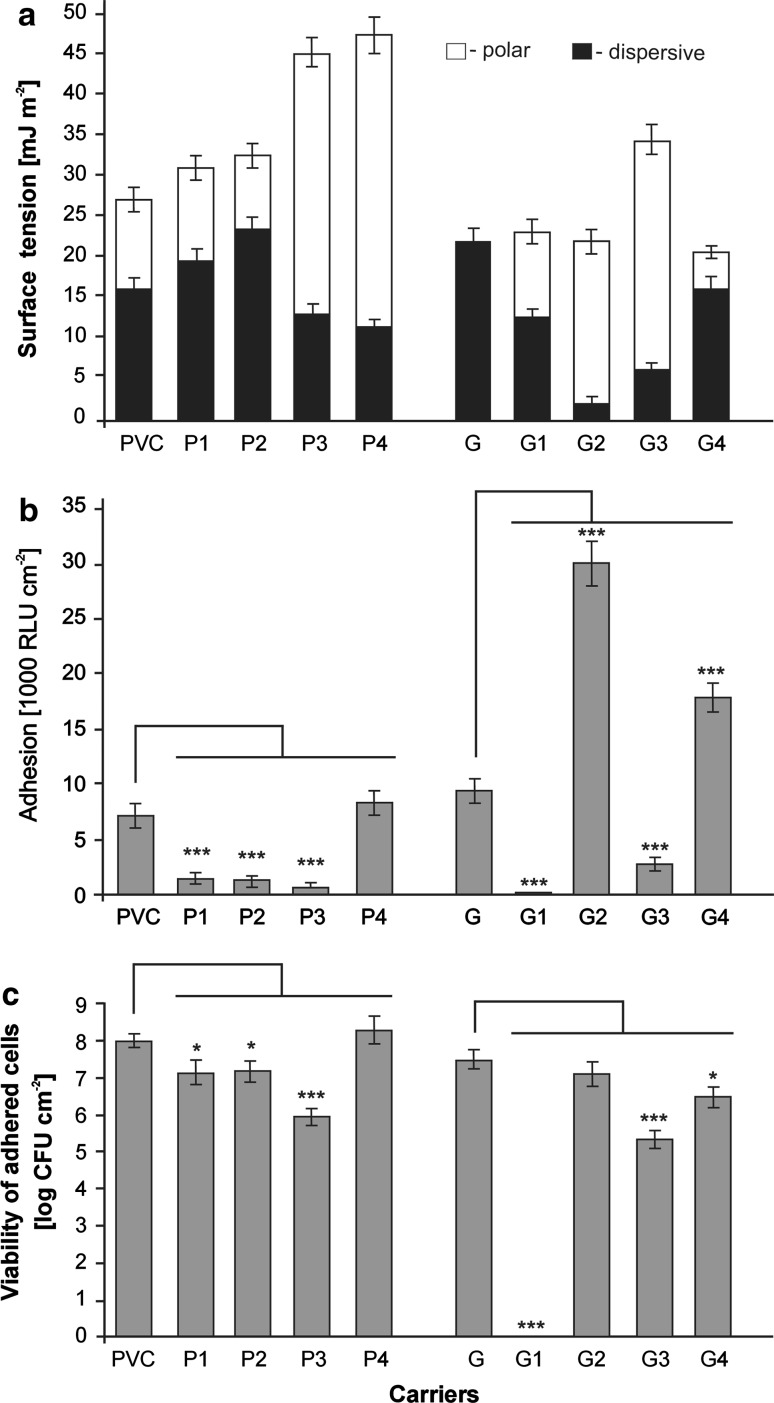
Surface characteristics. P: native PVC; P1: 1H,1H,2H,2H-Perfluorooctylmethyldimethoxysilane; P2: Isobutylmethyl dimethoxysilane; P3: (3-glycidoxypropyl)trimethoxysilane; P4: 3(1-Tertbutylamine-2-hydroxy)propyloxypropyl diethoxymethylsilane; G: native gumosil; G1: poly[dimethylsiloxane-co-(*N*,*N*-dimethyl-*N*-*n*-octylammoniopropyl chloride) methylsiloxane)] terminated with hydroxydimethylsilyl groups (20 %); G2: poly[dimethylsiloxane-co-(*N*,*N*-dimethyl-*N*-*n*-octylammoniopropyl chloride) methylsiloxane)] terminated with hydroxydimethylsilyl groups (10 %); G3: poly[dimethylsiloxane-co-(*N*,*N*-dimethyl-*N*-*n*-hexadecylammoniopropyl chloride) methylsiloxane)] terminated with hydroxydimethylsilyl groups (20 %); G4: poly[dimethylsiloxane-co-(*N*,*N*-dimethyl-*N*-*n*-hexadecylammoniopropyl chloride) methylsiloxane)] terminated with hydroxydimethylsilyl groups (10 %). **a** Surface tension; **b** Bacterial adhesion; **c** Number of viable bacterial cells on the tested surfaces. Data presented in **b** and **c** were analyzed by One-way ANOVA followed by Tukey’s multiple comparison test. *p < 0.05; **p < 0.01; ***p < 0.001 compared to native surfaces

Surface tension is one of the most important physicochemical properties of a solid surface. Figure 2a presents the results for surface tension measurements of the tested surfaces, i.e. the native materials and those after chemical modifications. The presence of active groups had an impact on the surface energy of the tested materials. The PVC surface modified with (3-glycidoxypropyl)trimethoxysilane (P3) and 3(1-Tertbutylamine-2-hydroxy) propyloxypropyl diethoxymethylsilane (P4) exhibited almost double the surface tension of the native PVC and of other modifications. We can observe that the presence of active groups resulted in a significant increase in the polar forces that contribute to the surface energy of the materials tested, especially P3, P4 and all gumosil modifications. However, no correlation between polar forces and luminometric results for adhesion (r = 0.08) or with viability of adherent cells (r = 0.14) was noted.

Throughout the 10-day incubation period, the bacterial attachment to the native and modified surfaces was assessed by luminometry. Figure 2b presents the results of the luminescence (in RLU cm^−2^) for the tested surfaces, which showed various levels of cell adhesion. For unmodified PVC and G surfaces, the RLU values were equal to 7,000 and 9,375 RLU cm^−2^, respectively. Significant differences (*p* < 0.05) were found by One-way ANOVA test in terms of the adhesion results (RLU cm^−2^) for the native G and all its modified surfaces: G1–G4. In the case of the PVC materials, significant differences were found in modifications P1–P3 but not in P4.

Better anti-adhesive properties were observed in the cases of P1, P2, and P3 modifications of the PVC, and of G1 and G3 surfaces of the modified gumosil. Especially the modification G1, with active part—*N*,*N*-dimethyl-*N*-*n*-octylammoniopropyl chloride (20 %), seemed to have the best antiadhesive features. For this modification a very low result equal to 1 RLU cm^−2^ was obtained.

The level of living bacterial cells on native surfaces after 10 days of incubation was 10^7^–10^8^ c.f.u. cm^−2^ (Fig. 2c). Almost all modifications with different organo-silanes contributed to a decrease in the number of adhered viable cells of *A. hydrophila*. The best antimicrobial properties were observed for gumosil modification G1. In this case, no viable cell per cm^2^ was detected.

Statistical analysis of the viability results for cells adhered to PVC materials confirmed that modification P4 did not exhibit the desired properties. Additionally, no significant differences between G and G2 surfaces were observed. The analysis of data for modified surfaces by post hoc Tukey’s test showed an absence of significant differences between P1 and P2 surfaces, both for adhesion and viability results. A significant correlation (r = 0.90) between the results from both analytical methods (luminometry and plate count) was found only in the case of the PVC surfaces. In the case of the gumosil the lower coefficient value (r = 0.65) was obtained.

**Fig. 3:**
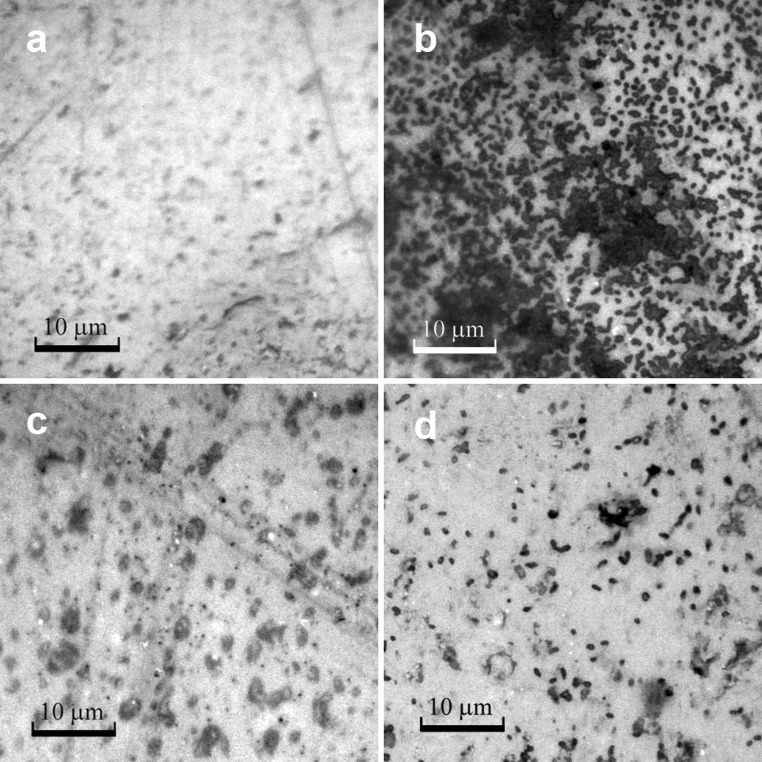
Adhesion of *A. hydrophila* to PVC surfaces after 10 days of incubation (at the same magnification, bars represent 10 μm). **a** Native surface before incubation; **b** Native surface after 10-day incubation in bacterial suspension; **c** Modified surface P3 before incubation; **d** Modified surface P3 after 10-day incubation in bacterial suspension

**Fig. 4:**
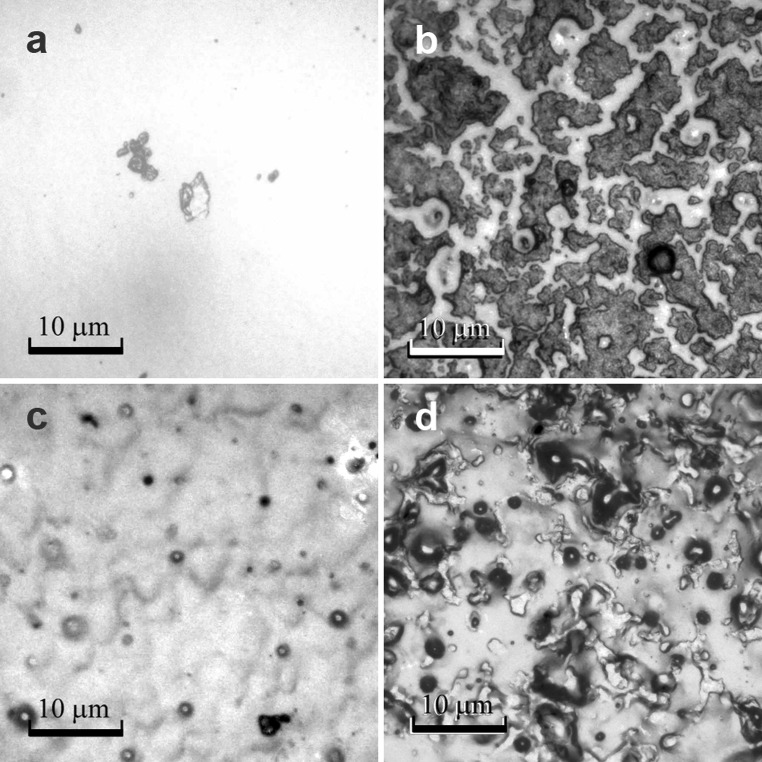
Adhesion of *A. hydrophila* to gumosil surfaces after 10 days of incubation (at the same magnification, *bars* represent 10 μm). **a** Native surface before incubation; **b** Native surface after 10-day incubation in bacterial suspension; **c** Modified surface G1 before incubation; **d** Modified surface G1 after 10-day incubation in bacterial suspension

Figures 3 and 4 show exemplary images of the native and modified PVC and silicone elastomer surfaces. The chemical modifications performed led to a significant reduction in the number of adhered *A. hydrophila* cells. Irregular cell adhesion on the surface was detected on each of the native materials. The heterogeneous nature of adhesion was observed especially on both native surfaces, resulting in a surface coverage ranging from approximately 30 to 50 % of the total area. However, the bacterial biofilms were differentiated structurally—single cells were visible only on the PVC surfaces, while on the gumosil and its modifications, the characteristic clusters were observed.

**Fig. 5:**
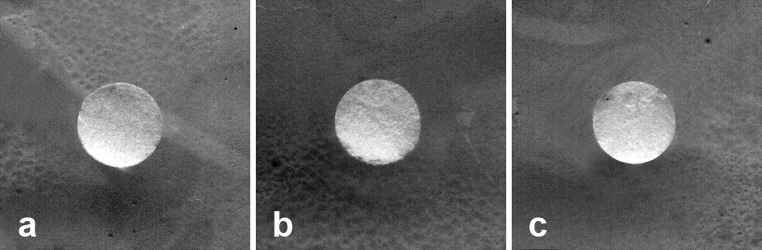
The antibacterial activity of the culture media after 10-day incubation with the carrier checked using the Bauer-Kirby method. **a** Native surface PVC; **b** Modified surface P3; **c** Modified surface G1

Based on the results, we can assume that the most effective antibacterial modification was obtained by 20 % of poly[dimethylsiloxane-co-(*N*,*N*-dimethyl-*N*-*n*-octylammoniopropyl chloride)methylsiloxane)] terminated with hydroxydimethylsilyl groups (G1) and (3-glycidoxypropyl)trimethoxysilane (P3), which were used for the modification of gumosil and PVC, respectively. In addition, the 10-day incubation of the carriers in the culture medium did not result in the occurrence of antibacterial compounds in concentrations able to inhibit cell growth (Fig. 5). These preliminary results, obtained by using the disk diffusion method, suggest that the antibacterial activity of active organo-silanes is associated only with the carrier.

## Discussion

A generalized relationship between surface tension and the level of adhesion was established as the ‘Baier curve’, the key feature of which is a minimal bioadhesion of around 22–24 mJ m^−2^. It was also found that surfaces with a critical surface tension of 20–30 mJ^−1^ m^2^ release diverse types of biofouling more easily than materials of higher or lower critical surface tension (Vladkova 2009; Li et al. 2010). All modified materials showed equal or higher surface tension values in comparison to the native surfaces. The values of this parameter ranged from 24.4 to 47.0 mJ m^−2^, with the lowest values noted for the native surfaces. The critical level 30 mJ m^−2^ was exceeded by all PVC surfaces and by modification G3. This may indicate that these materials are more adhesive than other surfaces. It is worth noting that the presence of active groups resulted in a significant increase in the polar forces contributing to the surface energy of the modified materials. This fact was particularly evident in the gumosil modifications. However, no correlation between polar forces and QAS concentrations in the gumosil matrix was found. Similar results were obtained by Fortuniak et al. (2011). This phenomenon may be explained by the fact that silanes can adopt various conformations or orientations at different polymer interfaces, diffuse through polymer or form interfacial hydrogen bonding (Chen 2012).

It is known that significant polar forces can alter the adhesion properties of solid materials (Gerberich and Cordill 2006). Additionally, any surface will become modified rapidly by immersion in natural waters and by adsorption of conditioning films (Vladkova 2009). In our study, the surfaces showed various surface tension values, but during the 10-day adhesion tests they were in contact with water containing a small amount of organic matter. This fact could have influenced the subsequent adhesive events associated with the attachment of bacterial cells.

The growth of *A. hydrophila* in chlorinated water has been related to water temperature, residual chlorine, and the interaction between these two variables (Pablos et al. 2009). A number of studies on low-nutrient environments show that *A. hydrophila* can remain viable for extended periods of time (Vivas et al. 2004). These experiments reveal that microgram-per-liter concentrations of amino acids and long-chain fatty acids promote the growth of aeromonads in water distribution systems. Environmental parameters also influence the adhesion process (Sautour et al. 2003). Bacteria in the natural environment are often found under nutrient-limiting conditions, and the colonization of solid surfaces has been described as the basic and natural bacterial strategy and survival mechanism (Cappello 2008). Therefore, research into the adhesion capacity of *A. hydrophila* cells in nutrient-limiting conditions appears particularly necessary. For this reason, in this study we used a poor culture medium, containing 200 mg l^−1^ of peptone only.

The presence of antimicrobial compounds on the modified surface can stimulate, after long exposure time, the transition of cells into the “viable, but non-culturable” (VBNC) state (Lee et al. 2007; Oliver 2010). The presence of VBNC cells has been clearly shown using alternative methods, but currently there is no technique available which can quantify the whole viable population accurately. Therefore, in this study on bacterial adhesion, a rapid and simple luminometric method was used, allowing us to estimate the quantity of the biological material on the test surfaces. This approach is based on bacterial ATP quantification and can be used not only to determine the total number of adhering cells, but also to evaluate the cell viability of bacteria that are not able to grow. This method and luminometers were used successfully in the studies on biofilms conducted by Hallam et al. (2001) and Szczotko and Krogulski (2010).

According to the literature, a significant number of techniques can be used to measure cell adhesion (Vesterlund et al. 2005). Conventional methods used to determine the number of adherent microorganisms are based on swabbing and plating. In the plate count method, a biofilm is mechanically disrupted, before being serially diluted, and finally colony forming units are determined. Both methods used, the conventional plate count and the unconventional luminometry, require scraping bacterial biofilm. This procedure is difficult because biofilms are resilient, adherent and quite resistant to stripping by swabs. Additionally, swabbing may give misleading results because of the difficulties in obtaining a homogeneous cell suspension. The use of various procedures supporting “disrupting” (chemicals, ultrasound, laser shockwaves) is usually laborious and may be associated with a reduction in cell viability (Oulahal et al. 2004; Benoit et al. 2010; Navarro et al. 2011). It is worth noting that the plate count technique in particular requires that bacteria should remain culturable after the release process. Due to the difficulty of obtaining a homogeneous suspension after swabbing, the additional analytical method of microscopy was used.

Depending on the type and structure of the surface of the biofilm, the results obtained by the luminometric and the plate methods may be more (polyvinyl chloride, r = 0.90) or less (gumosil, r = 0.65) closely correlated. Different correlation coefficients may be the result of the arrangement of the biofilm on the materials tested. The additional method of light microscopy allowed for accurate evaluation of cell loading. It was found that the bacterial biofilms were structurally differentiated—single cells were visible on the PVC surfaces, while on the gumosil materials the characteristic clusters were observed.

The inhibition of growth and adhesion of bacteria can be achieved by different chemical compounds covalently binding to the native surface (Rodrigues et al. 2006). The modification of PVC with four organo-silanes, containing different functional groups, gave the desired effects in three cases only (P1–P3). P1–P3 modifications contained reactive methoxy groups, and P4 surface-functional ethoxysilanes. According to the literature, methoxysilanes are generally reported to possess higher reactivity and toxicity compared to ethoxysilanes (Majumdar et al. 2011). Therefore, from the surface tension values and data from adhesion analysis, we can conclude that the incorporation of functional chemically-different groups into native material may yield different results.

In this study, the best modification proved to be the treatment of gumosil with poly[dimethylsiloxane-co-(*N*,*N*-dimethyl-*N*-*n*-octylammoniopropyl chloride) methylsiloxane)] terminated with hydroxydimethylsilyl groups (20 %). This compound containing a quaternary ammonium salt (QAS) inhibited the growth and adhesion of bacteria due to its high antibacterial and anti-adhesive properties. The biocidal QAS groups were commonly used for surface modifications (Li et al. 1998). For example, positively charged silica-QAS core–shell nanoparticles showed enhanced inhibition of the growth of *E*. *coli* and *S*. *aureus* (Song et al. 2011). Positive charge inhibits biofilm progression from the initial adhesion stage towards the growth stage, since immobilized QAS molecules interact with the cell membranes of adhering bacteria, presumably causing membrane leakage and cell death (Rodrigues et al. 2006). Nevertheless, it was documented that different chain-length QAS may give different adsorption properties and different antibacterial activities (Erkan et al. 2010; Fortuniak et al. 2011). It was confirmed that the molecules were not identical to the comparator and that subtle alterations, possibly due to structural differences, spatial orientation, and/or impurities, may have affected their antibacterial activity (Rodriguez et al. 2010). This fact was also observed in our study, and the best activity was noted in the shorter chain and the higher concentration of QAS (G1). Therefore, we may also assume that the antibacterial activity of the compound is also determined by the length and spatial orientation of its molecule.

The PVC and silicone elastomer surfaces modified with active organo-silanes showed various inhibition activities concerning the growth and adhesion of *A. hydrophila*. Changes in the total surface tensions and the introduction of chemically active compounds that can affect the surface seem to be the main reasons for this behavior. Finally, we can conclude that the activation of native surfaces by organo-silanes, especially by active QAS, may be a useful technique for the inhibition of *A. hydrophila* adhesion. Nevertheless, the potential application of active compounds in antibacterial materials requires further verification by long-term studies.
